# Development of a new wealth index for Tanzania: the moderated effect of the implementation of 1,7- malaria reactive community-based testing and response (1,7-mRCTR) by socioeconomic position (SEP) with malaria prevention

**DOI:** 10.1136/bmjgh-2025-021154

**Published:** 2026-01-07

**Authors:** Chen Gao, Sikai Huang, Haoyue Yin, Shenning Lu, Longsheng Liu, Yeromin P Mlacha, Prosper Chaki, Xiao-Nong Zhou, Ning Xiao, Sol Richardson, Duoquan Wang

**Affiliations:** 1 National Institute of Parasitic Diseases, Chinese Center for Disease Control and Prevention; Chinese Center for Tropical Diseases Research, Shanghai, China; 2 National Key Laboratory of Intelligent Tracking and Forecasting for Infectious Diseases, Shanghai, China; 3 Key Laboratory on Parasite and Vector Biology, Ministry of Health, Shanghai, China; 4 WHO Centre for Tropical Diseases, Shanghai, China; 5 National Center for International Research on Tropical Diseases, Ministry of Science and Technology, Shanghai, China; 6 Department of Epidemiology and Biostatistics, School of Public Health, Anhui Medical University, Hefei, Anhui, China; 7 Vanke School of Public Health, Tsinghua University, Beijing, China; 8 Environmental Health and Ecological Science Department, Ifakara Health Institute, Dar es Salaam, Tanzania; 9 School of Global Health, Chinese Centre for Tropical Diseases Research, Shanghai Jiao Tong University School of Medicine, Shanghai, China

**Keywords:** Malaria, Health policy, Public Health

## Abstract

**Introduction:**

The China-UK-Tanzania pilot project of 1,7-malaria reactive community-based testing and response (1,7-mRCTR) approach was implemented in Tanzania between 2015 and 2018. This project targeted villages with the highest malaria incidence to conduct screening and treatment. While socioeconomic factors are known to be strongly associated with malaria burden, their specific impacts on malaria prevention behaviours during the 1,7-mRCTR implementation period remained unclear. This study aimed to construct a household wealth index and investigate its association with malaria prevention outcomes within the context of 1,7-mRCTR.

**Methods:**

We used data from two cross-sectional household surveys conducted in 2015 (baseline) and 2018 (endline), covering 19 686 households. A 12-item wealth index was constructed using Mokken scale analysis, with weighted wealth scores calculated via multiple correspondence analysis to categorise households into wealth tertiles. Using logistic regression within a Difference-in-Differences (DID) framework, we assessed the association between household wealth and the household ownership of useful long-lasting insecticidal nets (LLINs), use of LLINs and use of antimalarial drugs.

**Results:**

Analysis of the pooled data showed that households in the first (poorest) tertile had significantly lower odds of owing LLINs (OR=0.62, 95% CI 0.54 to 0.70, p<0.001) and using LLINs (OR=0.53, 95% CI 0.45 to 0.62, p<0.001) compared to the third (wealthiest) tertile. The DID analysis, accounting for the interaction between the intervention period (2018 vs 2015) and wealth tertile, showed a significantly greater increase in the odds of owing LLINs (OR=1.26, 95% CI 1.03 to 1.56) and using LLINs (OR=1.88, 95% CI 1.25 to 2.82) among households in the first tertile compared with the third tertile.

**Conclusion:**

The wealth index effectively differentiated household socioeconomic status, revealing significant wealth-based disparities in malaria prevention behaviours. Importantly, the implementation of the 1,7-mRCTR approach appears to have had a disproportionately positive effect on poorer households, leading to a reduction in wealth-based inequalities related to key malaria prevention measures.

**WHAT IS ALREADY KNOWN ON THIS TOPIC**

- Currently, studies on the implementation of 1,7-malaria reactive community-based testing and response (1,7-mRCTR) mainly focus on its impact on malaria prevalence. Given that malaria is a poverty-related disease and that the effectiveness of 1,7-mRCTR implementation may vary across the population by socioeconomic position (SEP), this study analyses the relationship between SEP and malaria prevention measures by establishing a wealth index.

**WHAT THIS STUDY ADDS**

- The implementation of the 1,7-mRCTR approach has positively promoted mosquito net ownership and usage among poorer households.

**HOW THIS STUDY MIGHT AFFECT RESEARCH, PRACTICE OR POLICY**

- This study provides evidence that the 1,7-mRCTR approach has improved malaria control outcomes in Tanzania. Specifically, it enhanced household awareness of bed nets and demonstrated that this strategy had a more significant impact on economically disadvantaged populations. These findings offer valuable insights for future implementation of 1,7-mRCTR in other regions.

## Background

### Malaria burden in Africa

 Over the past decades, substantial progress has been achieved in reducing the global burden of malaria; however, in recent years, despite significant increases in financial and policy commitments towards control efforts, the decline in malaria morbidity and mortality has reached a plateau.^1 2^ Africa, which continues to bear the disproportionately largest share of the global malaria burden, has not seen a universally significant decrease in the transmission rate across the continent, especially in the southern and northwestern regions of Tanzania, where the intensity of transmission declines slowly.^3^ Current malaria control efforts in these regions rely on a suite of established interventions, such as insecticide-treated nets (ITNs), long-lasting insecticidal nets (LLINs), indoor residual spraying, artemisinin-based combination therapy, and malaria case management.^4^,^6^ While Tanzania has scaled up its nationwide malaria control programmes over the past few decades, expanding geographical coverage and enhancing prevention strategies to improve testing and treatment quality and accessibility, these initiatives still face substantial challenges, notably inadequate human, financial and material resources.^6^,^8^ With Tanzania ranking sixth globally for total number of annual malaria cases in 2024, a significant risk of malaria infection persists among the population.^1^

### 1,7-malaria Reactive Community-based Testing and Response (1,7-mRCTR) approach in Tanzania

Recognising the need for innovative approaches to sustain and accelerate progress towards malaria elimination, the WHO’s Global Malaria Technical Strategy 2016–2030 emphasises the transformation of malaria surveillance and response strategies into core intervention pillars.^9^ Leveraging robust surveillance data allows for the provision of more targeted interventions, and community-level testing facilitates rapid case detection, prompt treatment, and improved prevention of further transmission. It was in this context that the 1,7-mRCTR approach was first piloted as a collaborative China-UK-Tanzania pilot project in the Rufiji district of Tanzania between 2015 and 2018.^10 11^ This approach was inspired by China’s successful ‘1-3-7 norm’ for malaria elimination but was adapted and improved to suit the local conditions of medium to high-transmission settings prevalent in the pilot area.^12^,^14^ The 1,7-mRCTR approach mandates health facilities (HFs) to report any confirmed malaria cases within 24 hours (1 day) and to conduct follow-up community-wide testing using rapid diagnostic tests (RDTs) and treatment across the highest risk villages within 7 days (7 days) based on real-time surveillance data, to interrupt malaria transmission and *Plasmodium* life-cycle.^10 11 15^ In addition to community screening and treatment, the 1,7-mRCTR approach also includes quality control supervision of case detection ability by increasing the coverage of parasitological diagnosis among all suspected malaria cases in the corresponding community. Through community health education campaigns, it is recommended that all village members seek treatment at health institutions when experiencing febrile illnesses. Project activities included promotion of health education activities through easy-to-understand brochures and posters written in local languages. Crucially, the project team conducted extensive community mobilisation activities, such as community dramas, football matches, and distribution of T-shirts and banners with promotional messages, to enhance community acceptance of screening and treatment. Moreover, these intensive, high-visibility community activities, along with the frequent presence of community health workers (CHWs) and mobile testing stations, cultivated a strong ‘malaria control’ atmosphere in terms of increased knowledge and risk perception, enhanced community dialogue and norms, and building trust and promoting health service utilisation. This was also the first attempt to adopt a community-reactive surveilance and response strategy in high-prevalence environments. A previous study evaluating this pilot project demonstrated a significant decrease in malaria prevalence in the intervention wards, which fell from 26.0% (95% CI 23.7 to 28.4) at baseline to 4.9% (95% CI 4.0 to 5.9) at endline, representing an 81% reduction.^15 16^ Furthermore, this study also indicated an association between malaria incidence and asset ownership-based household economic status.^11^

### Socioeconomic position and malaria

Malaria is widely recognised as a disease of poverty, with over 90% of malaria cases and deaths concentrated in the poorest countries.^11 15 17 18^ Measuring socioeconomic position (SEP), defined as the aggregate of social and economic factors shaping an individual’s or group’s place within society, is critical.^19^ SEP serves both as a socioeconomic fundamental determinant of malaria risk and as a confounder in most observational malaria studies.^20 21^

Various studies have assessed differences in the impact of SEP on disease-related interventions in Africa, especially those related to economic inequality and health service inequality. A study using Demographic and Health Survey data from 25 sub-Saharan African countries revealed that a pro-rich bias in vaccination coverage, with greater wealth-related inequalities linked to household asset ownership in countries with lower overall vaccination rates. These inequalities disproportionately impacted disadvantaged groups, highlighting the role of socioeconomic factors in vaccine accessibility and uptake.^22^ Similarly, research on Directly Observed Treatment, Short course (DOTS), a short-term intervention measure for tuberculosis in Bangladesh found that the prevalence of tuberculosis in the lowest quartile is five times higher than that in the highest quartile. SEP was estimated by determining the household asset score, which is based on ownership of consumer goods related to wealth status. Although DOT testing and treatment itself are free, the treatment of tuberculosis lasts for a long time, and the transportation costs to the medical point, cost of missed work and possible informal costs constitute a huge obstacle. The lack of support during the transition period has had a serious impact on the poor.^23^ In a study that integrated three social marketing programmes, malaria, HIV and family planning, wealthy populations are more likely to have access to health interventions. All three measures indicated that ideal health outcomes are typically concentrated in the wealthier population.^24^ A cohort study on malaria in Uganda also showed that households with higher wealth index scores have a lower risk of malaria.^18 25^ These studies underscore the clear association between SEP and disease intervention effectiveness in Africa. At the household level, families with lower SEP may not be able to afford long-loasting insecticidal nets (LLINs), house screening or improved housing structures (such as concrete floors and iron roofs), which can effectively reduce mosquito contact. On the other hand, differences in levels of education result in varying knowledge about malaria prevention and treatment, as well as differences in seeking healthy behaviours. At the societal level, there are significant barriers for families to access healthcare, including transportation costs to medical facilities, loss of income due to seeking medical treatment (opportunity costs), and poor geographic accessibility. In addition, it also includes distrust of the healthcare system, and language or gender-related barriers.^26^ Understanding the socioeconomic characteristics of target populations is essential for malaria risk stratification and adjusting the provision of health services. Despite this known critical link, some large-scale surveys in Tanzania have not included comprehensive SEP indicators.^27 28^ Furthermore, in low- and middle-income countries (LMICs) like Tanzania, directly measuring income or consumption can be challenging and less reliable, necessitating use of proxy indicators.

Crucially, while previous studies focusing on the 1,7-mRCTR pilot project in Rufiji have primarily evaluated its overall effectiveness in malaria control, there has been limited attention paid to the potential role of SEP in determining which households benefit most from the innovative approach.^11 15^ This represents a significant gap in understanding how to maximise the equitable impact of such targeted interventions.

### Measuring SEP

SEP can be directly measured using household consumption, expenditure or income, or indirectly using proxy indicators such as wealth index, occupation, household vulnerability and education measures.^29^,^32^ In high-income countries, income is the main method for quantifying SEP; however, in many LMICs, income is not stable and can vary greatly due to time fluctuations, seasonal changes and economic instability. Additionally, most household consumption may come from savings or loans, and a lot of ‘income’ is not measured in monetary terms due to use of barter, or non-market acquisition of resources, so income may be an unreliable indicator of SEP.^2133^,^35^ Moreover, education in low-income countries targets social welfare dimensions that are different from financial indicators. Measurement of level of education may be influenced by methodological issues such as assumptions that each year of education is equally indicative of an increase in SEP and is of equal quality for each student. It is often more indicative of community-level social development than of household-level SEP.^36^ In Tanzania, there were few national-level surveys with wealth indices available. In household surveys in LMICs, asset indices are currently the most widely used method for quantifying SEP via indirect indicators.^35 37^ This is because investigators can quickly and objectively measure assets and maintain relative stability over time.^35^ Housing construction is also one of the indicators measured by SEP in Tanzania. As a household’s primary residence, a housing structure typically undergoes limited change over time. A study in rural Tanzania found that low-income levels prevented them from improving housing quality. Corresponding materials and energy sources were selected based on the household’s economic situation during the construction of the house.^38^ Many studies have estimated wealth index scores based on household assets and the risk analysis of malaria infection, indicating that the wealth index is a useful and reliable method for malaria risk assessment.^17 18 25^ Therefore, we attempted to investigate specific issues related to asset ownership, housing characteristics and materials in Tanzania during cross-sectional surveys.

This study aimed to construct a household wealth index based on asset ownership, housing characteristics and materials, using data from baseline and endline household surveys, within the context of the 1,7-mRCTR implementation in Tanzania between 2015 and 2018. Building on this wealth index, the study examines its association with household malaria prevention behaviours to understand the impact of household SEP on malaria control during the pilot project’s implementation.

## Methods

### Study setting

The 1,7-mRCTR was implemented as a pilot intervention in Tanzania from April 2015 to October 2018, covering one district with two control wards (Bungu and Kibiti) and two intervention wards (Chumbi and Ikwiriri) (the list of study villages is attached in online supplemental table 1). A distance of at least 30 km was required between the intervention and control wards to minimise potential contamination. This operational research study employed a quasi-experimental design, comparing outcomes in two separate, representative pilot communities. The goal of the project was to explore a locally-tailored model and mechanism on how Chinese antimalarial experiences could be adapted in local contexts to reduce the disease burden of malaria based on the existing local system. Intervention and control wards were matched based on historical malaria incidence ratios (MIRs) and test positivity rates from 3 years of health facility data preceding the intervention.^10^

### 1,7-mRCTR implementation

The operational planning and evaluation of malaria control activities for the 1,7-mRCTR approach was based on the lowest administrative geographical unit (village) corresponding to the patient’s residential address. The main intervention implemented in the intervention village was tailored to local conditions, guided by 1,7-mRCTR surveillance and treatment. These measures also included facilitating health education activities through easy-to-use booklets and posters written in local languages. The 1,7-mRCTR activities were exclusively implemented in the intervention villages. However, both intervention and control villages continued to receive equal access to the malaria control and prevention programmes delivered nationwide through the National Malaria Control Programme by the Ministry of Health.

The 1,7-mRCTR study team developed a case-based reporting system using the Open Data Kit (https:// opend atakit. org/) tool to capture information on malaria cases at HFs. The reporting system was compatible with the District Health Information Software 2 (DHIS2) platform (https:// www. dhis2 sympo sium. org/) and allowed data aggregation and sharing. We provided tablets to HFs to collect case-based data, including patients’ demographic information and their village of residence. Data from all confirmed malaria cases were aggregated weekly to calculate a village-level MIR. On identification of a village with the highest MIR, a group of CHWs established community-based mobile test stations in each hotspot village within the next week. RDTs (CareStartTM Malaria Pf/PAN (HRP2/pLDH) Ag Combo, Access Bio, Inc 65 Clyde Rd., Suite A, Somerset, NJ 08873, USA) were performed at community testing stations. A full regimen of artemisinin-based combination therapies dihydroartemisinin-piperaquine phosphate (DHA-PPQ) was provided to participants who tested positive for malaria.^39^ More detailed information regarding the specific operational procedures of the 1,7-mRCTR implementation can be found elsewhere.^10 11 15^

### Household survey data collection

Data were derived from two waves of cross-sectional household surveys: a baseline survey conducted from September to November 2015 and an endline survey conducted from March to April 2018. These surveys were administered to independent samples of households within both intervention and control wards.

Before study implementation (baseline survey in 2015), the latest baseline census data of study area were reviewed (with a total population of approximately 243 449 in selected communities) for determination of the number of households and age composition to be included in the sampling frame. All households were enumerated with a unique identification number for random sampling procedures. All households’ respondents (one household head and one competent adult household representative; for children who were unable to respond to the survey themselves, we interviewed the child’s caregiver on questions related to use of preventive measures and care seeking behaviours). In households that declined participation, nearby households with similar characteristics were surveyed as substitutes. A structured questionnaire was designed according to standard Roll Back Malaria’s Monitoring & Evaluation Reference Group (RBM-MERG) guidelines, collecting data on socio-economic characteristics, knowledge and usage of malaria prevention measures, health expenditures, health service usage and travel history, and more detailed information on the cross-sectional survey protocol can be found elsewhere.^40 41^

### Patient and public nvolvement

Informed consent was obtained from heads of household and household members who were 18 years of age or above. For those under 18 years of age, informed consent was obtained from parents or guardians. Institutional ethical approval was obtained from the Ifakara Health Institute Institutional Review Board (IHI/IRB/EXT/No: 18–2020) and the National Institute of Medical Research (NIMR/HQ/R.8a/Vol. IX/3634).

### Variables

#### Household wealth measure

A 12-item wealth scale indicating household SEP from a wide range of asset items and housing construction items was generated through an iterative multi-step process, to assign each household a weighted score based on the number of asset items present.


$$Score=\sum_{i=1}^{n}\left(1_{\left\{{\left\{item\right\}}_{i}=\;1\right\}}\times {\left\{score\right\}}_{i}\right)$$

Households were then categorised into three tertiles.

#### Outcome measures

We acquired information on 1,7-mRCTR implementation, households’ malaria prevention behaviour and malaria infection during two survey waves. Variables relating to 1,7-mRCTR implementation were Intervention (intervention/control), which was defined as household in 1,7-mRCTR target wards, and accepted all components of 1,7-mRCTR. Household survey, baseline or endline survey, was operationalised as a binary variable. Household malaria prevention behaviour was assessed through indicators including net ownership (yes/no), defined as the household having mosquito nets; useful LLINs (yes/no), defined as the household owns at least one LLIN, and that LLIN is within its useful lifespan as defined by the specific product’s expiration date; using LLINs (yes/no), was defined as the household respondent slept under useful LLINs the previous night preceding the survey.

Malaria infection outcomes included fever (yes/no), defined as the household respondent experiencing fever in the last 14 days; seeking treatment (yes/no), defined as household respondents seeking treatment for any source who had a fever; antimalarial drugs use (yes/no), defined as antimalarial drugs given to household respondents who had a fever and sought treatment at the facility. All outcomes were operationalised as binary variables.

#### Demographic variables

Demographic factors measured for each household across two survey waves included education of household head (any formal education, yes/no), gender of household respondent (male/female) and age group of household respondent (under 25/25–49/50–74/75 or above).

#### The conceptual framework of variables in 1,7-mRCTR

**Figure 1:**
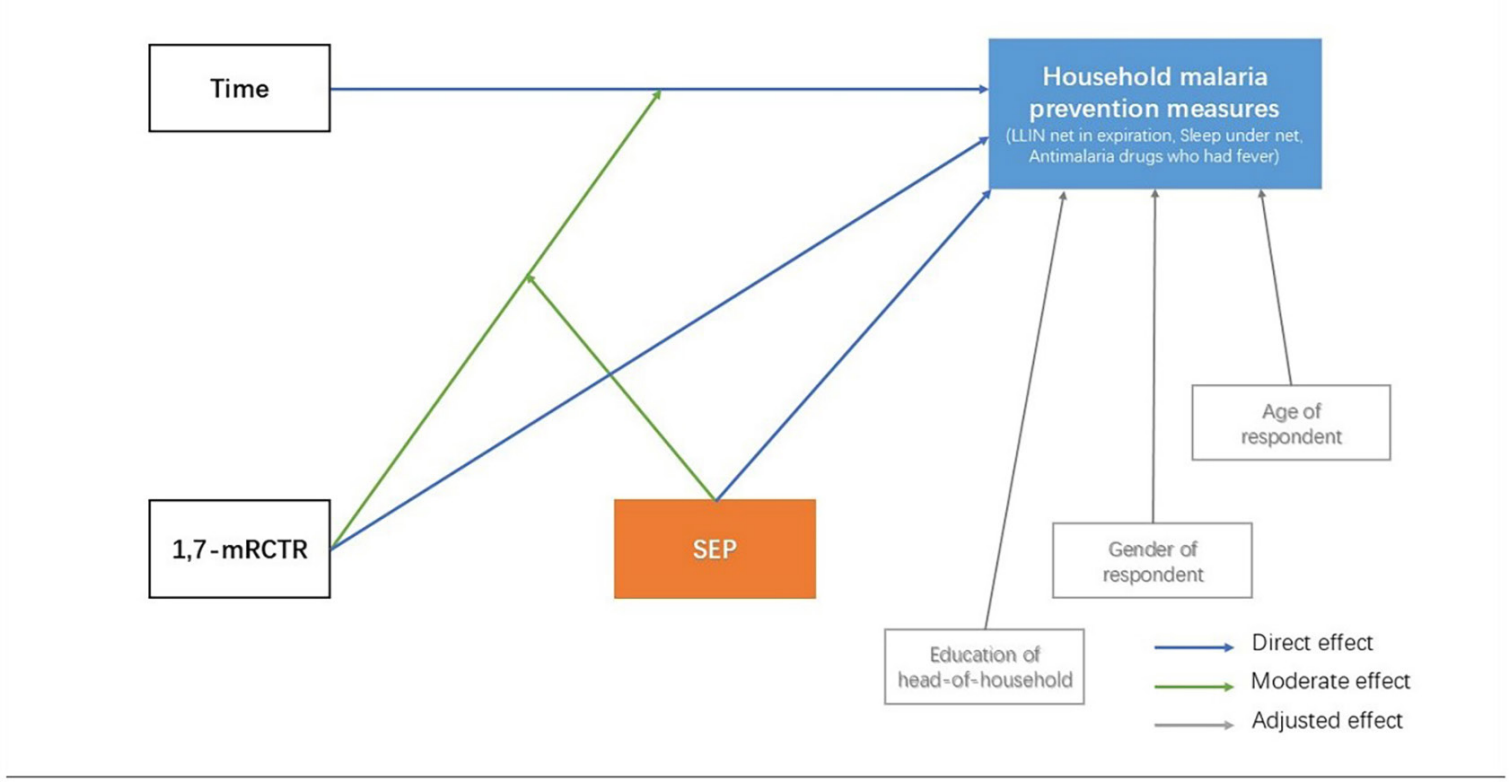
Diagrammatic representation of the conceptual framework of variables in 1,7-mRCTR (the grey part is the confounder, while orange and blue are our main study analysis outcomes). 1,7-mRCTR, 1,7-malaria reactive community-based testing and response; LLIN, long-lasting insecticidal net; SEP, socioeconomic position.

We hypothesised that SEP directly affects household malaria prevention measures in our study. SEP may be a direct influencing factor of household prevention measures, as household wealth is directly related to their malaria prevention measures. SEP may also serve as an interaction factor that affects the process of households receiving intervention within 1,7-mRCTR implementation. We also assumed a certain degree of confounding effect by variables at the household level (education of the head-of-household) and at the household respondent level (gender and age of household respondents) (figure 1).

### Statistical analysis

#### Development of household wealth index

Step 1 involved item selection and scale construction: data on 19 potential wealth items (has animal, bed, mattress, wardrobe, sofa, watch, iron, radio, bicycle, motorcycle, car/tractor, cooking fuel, water source, light source, toilet type, floor material, wall material, roof material, ceiling type; all coded as binary variables) were available from the two survey waves. We refined our item selection using Mokken scale analysis (MSA), combining the two survey waves. This method used Loevinger’s H coefficient to assess whether a set of items measures a single latent trait (in our study, ‘wealth’) and validates that these items form a hierarchical structure. This step ensured that the asset and housing characteristic items ultimately included in our wealth index collectively form a psychometrically robust and unidimensional scale. We performed MSA and applied a standard statistical cut-off (c>0.3). 13 variables (watch, wall material, radio, iron, toilet type, water source, light source, sofa, floor material, car/tractor, mattress, wardrobe, ceiling type) met this criterion, indicating that they effectively measured the underlying wealth construct. The other six variables (has animal, bed, bicycle, motorcycle, cooking fuel, roof material) were dropped due to low loadings. The floor materials have a significant impact on the SEP of households in the two surveys. Discussions with local collaborators suggested that this might be influenced by survey design factors, community-level clustering of certain housing improvements tied to economic cycles (eg, post-harvest) or potentially random error. To enhance the comparability of the wealth index across the two survey waves for the Difference-in-Differences (DID) analysis, we excluded ‘floor material’, resulting in a 12-item index that showed improved consistency in tertile distribution between baseline and endline.

Step 2 involved weight calculation: multiple correspondence analysis (MCA) is a data dimensional reduction technique similar to principal component analysis but employs a non-parametric item response theory approach using a monotone homogeneity model. The assumptions of this model are unidimensionality, monotonicity and local independence.^42^ MCA is an effective technique for analysing non-parametric data and is particularly appropriate for the coded binary responses in our study. We used MCA to obtain the weights corresponding to items and then used them to calculate a composite wealth score for each household. Finally, households were then categorised into three tertiles from low wealth score to high wealth score.

#### Association of household wealth index with household malaria prevention outcomes, and malaria infection outcomes during 1,7-mRCTR

We described socioeconomic and demographic characteristics of study respondents (household and respondent) in intervention and control wards in two survey waves using numbers and percentages. χ^2^ tests were performed to assess the variable balance between intervention and control wards in two survey waves. We assessed the association between wealth index, operationalised as tertiles, and household mosquito net ownership using χ^2^ tests.

We used logistic regression models, within a DID framework, to assess the potential moderating effect of household wealth on the intervention’s impact; the models included the study arm (Intervention vs Control), Survey Wave (Endline vs Baseline), wealth tertile (using dummy variables with the third tertile as reference) and interaction terms. The core DID effect was captured by the interaction between Study Arm and Survey Wave (Arm×Wave). The moderating effect of wealth was assessed by including interactions between Wealth Tertile and the Arm×Wave term (eg, Wealth Tertile×Arm×Wave). The DID design compared the changes in outcome variables before and after 1,7-mRCTR implementation between the ‘intervention’ and ‘control’ areas by fitting a two-way interaction term between survey wave and allocation to the intervention or control groups. A key assumption of the DID method is that of parallel trends, such that in the absence of intervention, the changes in outcomes in intervention areas will be similar to those in control areas.^43^ Theoretical and empirical research indicates that the parallel trends assumption is more likely to hold when the two groups are more similar at baseline.

First, missing data were present in the observational dataset (the random distribution of missing data is shown in online supplemental table 2). Prior to performing logistic regression, we employed multiple imputation (MI) (10 imputations) using the mi command in Stata to handle missing observations through multivariate imputation.^44 45^ The analysis pooled results across imputed datasets for model refitting. The MI approach assumed missing-at-random (MAR) mechanisms based on the observations.^46^

Second, to improve its plausibility by reducing imbalance in the characteristics of the analytic sample across two groups in baseline survey to reduce potential confounding, we employed inverse probability of treatment weighting (IPTW), a generalised form of propensity score matching.^47^ First, we developed a logistic regression model to predict the probability of each household being assigned to the intervention group (ie, the propensity score), using all observable baseline covariates (educational level of the household head, the respondent’s age and sex). Second, we calculated a weight for each household, which is the inverse of the probability of receiving the treatment it actually received (intervention or control). Online supplemental table 1 provides a detailed description of the weighted proportions, t statistic values and p values of all baseline covariates between the intervention group and the control group after applying IPTW weights.

Finally, we applied these weights to the DID analysis. We used logistic regression to fit the models (calculating adjusted odds of having useful LLINs, using LLINs and antimalarial drugs, with 95% CIs, p value, Pseudo-R² and AIC for logistic models). We fitted four models with difference-adjusting variables for three outcomes. Model l included only time, intervention and the interaction of time and intervention. Model 2 added the SEP and the three-way interaction of time, intervention and SEP. Model 3 controlled for household characteristics (education of the head of the household). Model 4 controlled for household characteristics (education of the head of the household) and household respondent characteristics (member age and member gender). We used the margin command to calculate the average marginal effect of interventions and survey time points, and to explain the combined effect in a more interpretable way by predicting the margin (online supplemental table 4) demonstrates the combined effects of interventions and time in DID.

Data were analysed using Stata V.17.0. Mokken scaling analysis was conducted with the ‘msp’ package.

## Results

### Socioeconomic and demographic characteristics of study participants by intervention allocation and survey waves

Online supplemental tables 5 and 6 present the socioeconomic and demographic characteristics of study households in intervention and control wards across two survey waves. Overall, this study included 9552 participants (4681 in the intervention; 4871 in the control) from the baseline survey and 10 134 participants (4560 in the intervention and 5574 in the control) from the endline survey.

At baseline, the proportion of households in the first tertile (poorest) intervention (38.41%) versus control (36.85%) was significantly higher than the third tertile (wealthiest) intervention (31.10%) versus control (28.17%), and this difference was statistically significant (p<0.001). Similar statistically significant differences in wealth tertile distribution between study arms persisted in the endline survey.

Compared with households in control wards, a significantly higher proportion in the intervention wards owned mosquito nets (83.94% vs 69.70%, p<0.001). The proportion of households in intervention wards with useful LLINs was also significantly higher (56.12% vs 43.88%, p<0.001) than that in control wards in baseline survey. A high proportion of respondents slept under a net last night (66.39% vs 33.61%, p<0.001) in intervention wards in the baseline survey, and a higher proportion of respondents slept under a net last night (88.01% vs 11.99%, p<0.001) in intervention wards in the baseline survey.

Although there were no significant differences in the endline survey, the proportions of households owning mosquito nets (91.79% vs 76.68%), useful LLINs (68.03% vs 48.44%) and respondents using LLINs (88.01% vs 60.00%) were higher than the baseline survey. Over 50.00% of household respondents were male, and over 70.00% of respondents across the two waves were under 50 years.

At baseline, a higher proportion of household respondents had fever in the intervention wards (11.37% vs 9.01%, p<0.001), and the proportion of individual respondents receiving antimalarial drugs given at HFs in intervention wards was significantly higher than in control wards (65.02% vs 46.98%, p<0.001). Conversely, the proportion of individual respondents who reported fever in intervention wards was significantly lower than that in control wards (8.18% vs 9.56%, p=0.015) in the endline survey. The proportion of respondents who had a fever (11.37% vs 8.94%) in the endline survey decreased, but the proportion of seeking treatment from HFs (60.11% vs 68.24%) and obtaining antimalarial drugs (57.60% vs 14.71%) wss lower than in the baseline survey (online supplemental tables 5 and 6).

### Distribution of household ownership of asset items

**Figure 2:**
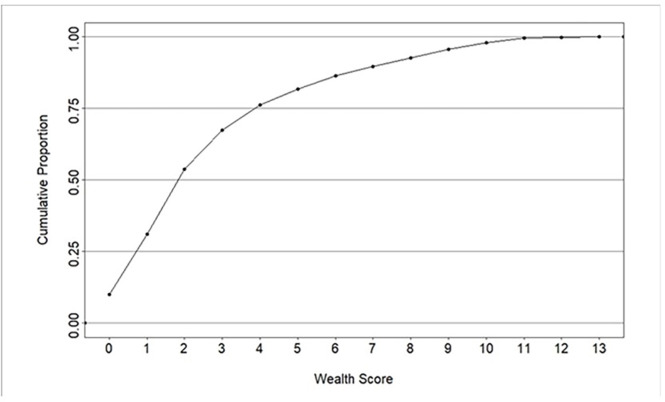
A cumulative proportion plot of the distribution of wealth score based on household ownership of assets and housing construction.

Figure 2 shows the cumulative proportion of the unweighted total score by household sample based on ownership of the 13 items. Household unweighted total score of one accounted for over 25%. Household unweighted total score of two accounts for over 50%. Household unweighted total score of four accounted for over 75%.

Table 1 presents a descriptive analysis of household wealth items in intervention and control wards in baseline survey and endline survey. Of 19 items analysed using MSA with a Loevinger coefficient of 0.3, the automated item selection procedure identified seven items related to personal use ownership (mattress, wardrobe, sofa, watch, iron, radio, car, tractor) and six items belonging to the category of housing quality (water source, light source, toilet type, floor material, wall material, ceiling type) belonging to one scale. One asset item was defined as household ownership or exclusive household access to a vehicle. Single-item Hi of each of the 13 items had values greater than 0.4, indicating ‘medium’ scale values. We focused on 13 items contributing to scale one.

**Table 1:** Item weights for calculating total scores using MCA analysis

Wealth items	Baseline survey (n=9552, %)	Endline survey (n=10 134, %)	Weight score
Watch	2137 (22.37)	1.066 (10.52)	2.384
Ceiling type	752 (7.87)	1.769 (17.46)	2.566
Wall material	1044 (10.43)	2.004 (19.78)	2.625
Radio	5376 (56.28)	5.271 (52.01)	1.396
Iron	1778 (18.61)	1.760 (17.37)	3.014
Toilet type	846 (8.46)	1.657 (16.35)	3.469
Water source	298 (3.12)	402 (3.97)	3.924
Light source	1818 (19.03)	3028 (29.88)	2.946
Sofa	1584 (16.58)	2152 (21.24)	3.403
Floor material	2899 (30.35)	4866 (48.02)	2.176
Car/tractor	282 (2.95)	170 (1.68)	3.563
Mattress	7143 (74.78)	9077 (89.57)	1.705
Wardrobe	1505 (15.76)	1965 (19.39)	3.394

MCA, multiple correspondence analysis.

The total situation of household ownership of wealth items in intervention wards in the endline survey was lower than that in control wards. Except for the four items of watch, radio, iron and car/tractor, the proportion of ownership of other items in households at the endline survey was higher than the baseline survey.

The items were weighted using the loadings from MCA from the first dimension according to MCA analysis, which explained 90% of the variance in the presence of scale items. Use of weights ensured that each wealth scale was evaluated based on its adjusted item scores.

Table 2 presents the changes in household wealth index ranks when excluding wealth items with higher weight. We summed the weighted scores from the 13-item for each household and constructed a wealth index by tertiles; the first tertile indicated the poorest households and the third tertile indicated the wealthiest. Specifically, 51.08% of households comprised the first tertile, 21.02% of the second tertile and 27.90% of households the third tertile, in the baseline survey. 36.11% of households comprised the first tertile, 26.53% of households the second tertile, and 37.36% of households the third tertile in the endline survey.

**Table 2:** Changes in household wealth index rank and with exclusion of wealth items

Excluded items	Baseline survey (n=9552, %)			Endline survey (n=10 134, %)		
	First tertile (poorest)	Second tertile	Third tertile (wealthiest)	First tertile (poorest)	Second tertile	Third tertile (wealthiest)
None	4879 (51.08)	2008 (21.02)	2665 (27.90)	3659 (36.11)	2689 (26.53)	3786 (37.36)
Ceiling type	5002 (52.37)	1752 (18.34)	2798 (29.29)	4040 (39.87)	2339 (23.08)	3755 (37.05)
Wall material	4939 (51.71)	1792 (18.76)	2821 (29.53)	3770 (37.20)	2673 (26.38)	3691 (36.42)
Toilet type	4908 (51.38)	1819 (19.04)	2825 (29.57)	3730 (36.81)	2667 (26.32)	3737 (36.88)
Light source	4978 (52.11)	1719 (18.00)	2855 (29.89)	3869 (38.18)	2609 (25.75)	3656 (36.08)
Floor material	3593 (37.62)	3131 (32.78)	2828 (29.61)	3013 (29.73)	3441 (33.96)	3680 (36.32)

Due to the significant differences in household wealth items between baseline and endline surveys, wealth items with p<0.001 and higher weights were selected for exclusion one by one. When excluding wealth items with higher weights one by one, only when floor materials are excluded from the SEP score, the gap in the distribution of the overall household wealth index between two surveys narrowed, especially in the change of the first tertile and second tertile ratios. Therefore, the wealth index we used in the subsequent regression analysis included just 12 items (excluding floor material).

Table 3 presents the distribution of ownership and use of LLINs in households by their wealth index. Household ownership of mosquito nets was inversely associated with the wealth index. Among households with nets, 2.09% did not have LLINs, and the proportion past expiration proportion was 25.38%. For use of LLINs by households in the study, the usage proportion of third tertile households was significantly higher than the first and second tertiles.

**Table 3:** Distribution of ownership and household use of LLINs and their wealth index

Variable	Wealth index (n=19 686, %)			Total
	First tertile (poorest)	Second tertile	Third tertile (wealthiest)	
	(n=6606, %)	(n=6572, %)	(n=6508, %)	
No net	1665 (25.20)	1043 (15.87)	352 (5.41)	3060 (15.54)
No LLINs	77 (1.17)	104 (1.48)	230 (3.53)	411 (2.09)
LLINs but past expiration	1676 (25.37)	1812 (27.57)	1508 (23.17)	4996 (25.38)
Useful LLINs	3188 (48.26)	3613 (54.98)	4418 (67.89)	11 219 (56.99)

LLINs, long-lasting insecticidal nets.

### Association of household wealth index with household malaria prevention and malaria treatment during 1,7-mRCTR

Online supplemental table 7 presents the regression results from the logistic regression models applying the DID framework to assess the association between household wealth tertile and the primary malaria prevention outcomes (useful LLINs ownership, using LLINs), and antimalarial drugs, considering the impact of the 1,7-mRCTR intervention.

#### Household malaria prevention outcomes

There were four DID models with different sets of covariates. Model 3 adjusting for wealth index and household covariates showed a statistically significant lower odds of net ownership in the first tertile compared with the third tertile (OR=0.43, 95% CI 0.28 to 0.48, p<0.001), but, based on the three-way interaction term, a statistically significant 45% increase in the odds of net ownership in the first tertile compared with the third tertile (OR=1.45, 95% CI 1.08 to 1.94, p=0.014) under 1,7-mRCTR implementation. Similar findings in Model 4 showed a statistically significant lower odds of respondents using LLINs in the first tertile compared with the third tertile (OR=0.44, 95% CI 0.37 to 0.52, p<0.001), and a statistically significant 214% increase in the odds of respondents using LLINs in the first tertile compared with the third tertile (OR=3.14, 95% CI 1.94 to 5.08, p<0.001) under 1,7-mRCTR implementation. The same effect size was observed when comparing the second tertile with the third tertile in terms of LLIN use (OR=1.63, 95% CI 1.04 to 2.54, p=0.031) under 1,7-mRCTR implementation.

Model 3 results showed that households in 1,7-mRCTR intervention wards had significantly higher odds of net ownership compared with control wards (OR=1.79, 95% CI 1.62 to 1.99, p<0.001). Compared with households that only participated in the baseline survey, those that completed the endline survey showed significantly higher odds of net ownership (OR=2.42, 95% CI 2.15 to 2.74, p<0.001). However, when considering the combined effect of the 1,7-mRCTR intervention and time, these endline households demonstrated significantly lower odds of net ownership compared with other households (OR=0.48, 95% CI 0.37 to 0.62, p<0.001). Model 4 results also showed the same finding for use of LLINs.

#### Malaria treatment outcome

Model 4 results suggested a 24% decrease in the odds of respondents receiving antimalarial drugs from HFs in the first tertile relative to the third tertile (OR=0.76, 95% CI 0.45 to 1.29, p=0.308), and a 58% increase in the odds of respondents receiving antimalarial drugs from HFs under the combined effect of intervention of 1,7-mRCTR and time in the first tertile relative to the third tertile (OR=1.58, 95% CI 0.22 to 11.58, p=0.652); these associations were not statistically significant, however.

## Discussion

Monitoring and surveillance are crucial in malaria-endemic areas to detect any increase in malaria cases, track potential outbreaks and evaluate interventions' effectiveness.^9^ The China-UK-Tanzania trilateral cooperation has initiated new pilot projects to optimise the surveilance tool in Tanzania.^10 12^ By sharing the ‘1-3-7 norm’, concept and practices into the surveillance response strategy, 1,7-mRCTR suitable for areas with high malaria transmission has significantly reduced malaria prevalence as well as the incidence rate.^11 15^ Previous studies have shown that there is a significant impact of SEP on outcomes for infectious disease interventions in Africa, with several studies highlighting the association between SEP and malaria prevalence and prevention behaviours.^18 25 34^ Despite this, there has been a notable lack of research specifically analysing the relationship between household SEP and implementation of the 1,7-mRCTR approach. This study addresses this gap by constructing a household wealth index—the first to our knowledge in this specific context—to measure household SEP in the Rufiji pilot area and investigate its association with key malaria prevention outcomes within the 1,7-mRCTR framework. Our findings provide valuable evidence to inform policy aimed at enhancing the equity and effectiveness of future intervention strategies.

### Summary of key findings

This study stratified the SEP of households in the intervention wards and the control wards through 12 wealth items. The study suggests that there are significant wealth differences in household malaria prevention behaviour. Households with the lowest wealth index were less likely to own LLINs and less likely to use LLINs the night before the survey. The richest households are more likely to use mosquito nets the night before the survey.

### Construction of a aocioeconomic index

Socioeconomic factors are an important influencing factor for malaria in Tanzania. Wealth indices have been widely used to quickly measure SEP in LMICs.^44^,^46^ Our study supports the utility of asset ownership and housing characteristics as relatively stable and objective indirect indicators of household SEP in this context.^37 48 49^ Furthermore, housing quality, a component of the wealth index, is directly linked to malaria risk as better-quality housing can reduce mosquito entry and breeding sites.^50^,^52^ Previous studies comparing indicators of socioeconomic inequality to measure malaria risk have found that wealth indices are a reasonable alternative to consumption in rural Tanzania.^34^

The study used the wealth index that excluded floor materials. While this addressed a methodological challenge for the current analysis, further investigation into the dynamics and measurement of housing improvement items is warranted in future research.

### SEP effect with household malaria prevention behaviour

Large-scale distribution campaigns and continuous distribution models are key strategies for increasing ITN coverage in Tanzania, which received a large proportion of globally distributed insecticide-treated nets (ITNs) between 2014 and 2016.^53^ However, mosquito nets, as consumables, need to be replaced regularly to ensure better capability of malaria prevention, and there are various missed populations that require additional efforts to improve or maintain the malaria prevention capability of mosquito nets. Our findings indicate that 95% of wealthier households have mosquito nets, and 57% of these households have useful LLINs in two surveys, which enable them to better prevent mosquito entry and bites during the use of mosquito nets. Other studies have found that wealthy urban households are more likely to own purchased mosquito nets.^54 55^ There is a stronger preference for specific mosquito net characteristics and sufficient resources to purchase mosquito nets. A study in Tanzania found that richer households are willing to pay for mosquito nets that have been treated with insecticides, are larger in size, and have other features lacking in public sector provided mosquito nets.^56^ Richer households may utilise ITNs with higher active ingredient concentrations, as well as timely replacement of damaged or ineffective nets. Matching the number of LLINs according to household size is also more common in wealthier households, allowing household respondents to sleep in mosquito nets to prevent mosquito bites. The impoverished population has always been a vulnerable group in terms of accessing medical services, lacking corresponding economic resources and channels to obtain medical and health services.^25^ This pilot project was conducted at the community level, promoting households within the research area. For impoverished households and remote or rural areas, there is a persistent and severe disparity in accessing healthcare services.

In terms of interpretation of model results, Model 3 shows that for both ownership and usage of LLINs, odds of these outcomes were significantly higher in the intervention group at baseline, and that these increased in both intervention and control group households between baseline and endline. While in absolute terms, the results of the descriptive analysis show a higher proportion of respondents in the intervention group owning and using LLINs at endline than in the control group, the relative increase shown by model results was less for the former; this finding may be explained by the intervention group starting from a significantly higher baseline and the occurrence of a ‘ceiling effect’, commonly observed in malaria^57 58^ and other^59^ interventions in settings comparable to Tanzania. At the same time, compared with the wealthiest households, the coefficient of the three-way interaction terms shows that, in relative terms, the poorest households of intervention wards in the endline survey had 1.26 times greater odds of owning mosquito nets, while odds of usage of LLINs was 1.88 times higher. This indicates a significant relative increase in the likelihood of poor households owning and using mosquito nets in the 1,7-mRCTR intervention wards in the intervention group, suggesting the intervention’s particular impact in this group and potential for reducing disparities in malaria prevention. Our results show that the 1,7-mRCTR approach promotes ownership and use of mosquito nets in households with low wealth index to a greater extent than those with higher household wealth. In addition, previous studies have observed that the prevalence of malaria in households in the intervention wards has significantly decreased compared with the baseline survey. While on the one hand, 1,7-mRCTR has achieved the effect of reducing the incidence rate of malaria, it is possible to prevent malaria by improving the possession and use of mosquito nets. Therefore, the LLIN distribution strategy should increasingly emphasise the stratification and localisation of LLIN distribution within specific subgroups, to strategically deploy LLINs based on infection risk.^60^ This study indicates that compared with control wards, there was a significant increase in the likelihood of poor families seeking treatment for fever and taking antimalarial drugs in 1,7-mRCTR intervention wards, with 1.64 times higher odds than that of affluent families, although the results are not significant. This may be because wealthy families prioritise their health, and regardless of whether 1,7-mRCTR is implemented, their household respondents will actively seek treatment and take corresponding medications when they have a fever. However, household respondents of impoverished households are less likely to attend health facilities for diagnosis after a fever, making it impossible to prescribe the right medicine for fever caused by malaria. After intervention with 1,7-mRCTR, free treatment was provided to individuals with fever, and DHA-PPQ was used to treat malaria patients. This is consistent with many research findings that higher wealth index scores among malaria patients are generally associated with better acceptance of antimalarial drugs.^61 62^ This study suggested that 1,7-mRCTR has played a more active role in both preventing and treating malaria among impoverished populations.

### Implications for policy

Building on previous findings demonstrating the effectiveness and feasibility of Tanzania’s 1,7-mRCTR approach as a surveillance-driven intervention, our study provides critical insights into its equity implications.^10 11 15^ The 1,7-mRCTR approach, with its emphasis on micro-stratification at the community level based on surveillance data, enables more rational allocation of resources and targeting of intervention measures. Our findings strongly emphasise the need to prioritise efforts that reduce the wealth-related gap in malaria prevention measures.

Despite ongoing efforts through mass campaigns and free distribution, challenges in supply chain, logistics, monitoring and public knowledge persist, contributing to unequal coverage and effectiveness.^63 64^ Increased investment in fundamental prevention measures is necessary to support the uptake and sustained use of interventions across different SEP groups. Within the 1,7-mRCTR framework, health promotion efforts could specifically target improving mosquito net use at the household level. The provision of free malaria drugs at community testing stations, as implemented in 1,7-mRCTR, could also play a role in narrowing the treatment gap by reducing financial barriers, although this requires further confirmation. Overall, tailoring interventions based on socioeconomic vulnerability, identified through tools like the wealth index, is essential for achieving equitable and effective malaria control.

### Strengths and limitations

The asset-based index provides a valuable tool for stratifying household SEP in LMIC settings. It can be quickly used for similar household surveys in Tanzania and other comparable settings. The advantage is that it accounts for the possibility that the housing construction may partially represent household SEP, which is also included in the SEP measurement of this study.

This study has some limitations. First, we conducted a study using household data from two cross-sectional surveys, and the results showed shortcomings in terms of temporal and spatial coverage. On temporal coverage, the different seasonal timings of the two cross-sectional surveys may have an impact on the transmission of malaria, and on spatial coverage, the pilot project covered just one district, Rufiji, to include the intervention group and the control group, which may limit the generalisability of our findings. Second, during the study design phase, the selection of intervention and control areas was primarily matched based on historical malaria incidence and health service accessibility. Our study is a secondary analysis of existing data from two surveys for evaluating the impact of 1,7-mRCTR on malaria outcomes and not purposively designed for this analysis; consequently, we observed disparities in wealth between the two groups at baseline.

## Conclusion

The wealth index we constructed has the potential to differentiate populations with different SEP to optimise intervention measures in the context of implementing 1,7-mRCTR. 1,7-mRCTR has improved households’ awareness of nets (ownership and using) and played a greater role in promoting malaria prevention among populations with lower wealth index. Although this is not the primary goal of the 1,7-mRCTR itself, improvement of household SEP and balanced access to malaria prevention resources can improve the malaria control environment. Our findings indicate that the community-based, proactive model of 1,7-mRCTR, which reaches deep into households, successfully overcame many of the barriers that typically prevent impoverished populations from accessing health services, thereby promoting health equity, and can aid understanding of the potential benefits and challenges of this innovative model implementation in similar settings.

## Supplementary material

10.1136/bmjgh-2025-021154 online supplemental table 1

## Data Availability

Data may be obtained from a third party and are not publicly available.
