# Supplementary material for: Development of a new wealth index for Tanzania: the moderated effect of the implementation of 1,7- malaria reactive community-based testing and response (1,7-mRCTR) by socioeconomic position (SEP) with malaria prevention
Source: BMJ Glob Health. 2026 Jan 7;11(1):e021154. doi: 10.1136/bmjgh-2025-021154 (PMC12781976; doi:10.1136/bmjgh-2025-021154)
Supplement: online supplemental table 1 [file bmjgh-11-1-s001.docx]

Supplemental Table 1. List of the 1,7-mRCTR study village in, Rufiji district, Tanzania.

| Study District | Survey | Control/Intervention | Study Ward | Study Village |
| --- | --- | --- | --- | --- |
| Rufiji | Baseline | Control | Bungu | Bungu A |
|  |  |  |  | Bungu B |
|  |  |  |  | Msafiri |
|  |  |  |  | Nyambili |
|  |  |  |  | Nyambunda |
|  |  |  |  | Pagae |
|  |  |  | Kibiti | Kibiti A |
|  |  |  |  | Kibiti B |
|  |  |  |  | Kibiti center |
|  |  |  |  | Kibiti south |
|  |  |  |  | Kingwila |
|  |  |  |  | Lumiozi |
|  |  |  |  | Makaoni |
|  |  |  |  | Mbebetini |
|  |  |  |  | Mwangia |
|  |  |  |  | Nyatanga |
|  |  |  |  | Sokoni |
|  |  |  |  | Zimbwini |
|  |  | Intervention | Ikwiriri | Ikwiriri center |
|  |  |  |  | Ikwiriri north |
|  |  |  |  | Ikwiriri south |
|  |  |  |  | Mgomba center |
|  |  |  |  | Mgomba north |
|  |  |  |  | Mgomba south |
|  |  |  |  | Umwe center |
|  |  |  |  | Umwe north |
|  |  |  |  | Umwe south |
|  |  |  | Chumbi | Chumbi A |
|  |  |  |  | Chumbi B |
|  |  |  |  | Chumbi C |
|  |  |  |  | King‘ongo |
|  |  |  |  | Kiwanga |
|  |  |  |  | Muhoro Magharibi |
|  |  |  |  | Muhoro Mashariki |
|  |  |  |  | Ndundutawa |
|  |  |  |  | Shela |
|  | Endline | Control | Bungu | Bungu A |
|  |  |  |  | Bungu B |
|  |  |  |  | Kimbuga |
|  |  |  |  | Kinyanya |
|  |  |  |  | Mangombela |
|  |  |  |  | Miwaga |
|  |  |  |  | Mng'aru |
|  |  |  |  | Msafiri |
|  |  |  |  | Nyambili |
|  |  |  |  | Nyambunda |
|  |  |  | Kibiti | Kibiti A |
|  |  |  |  | Kibiti B |
|  |  | Intervention | Ikwiriri | Ikwiriri center |
|  |  |  |  | Ikwiriri north |
|  |  |  |  | Ikwiriri south |
|  |  |  |  | Mgomba center |
|  |  |  |  | Mgomba north |
|  |  |  |  | Mgomba south |
|  |  |  |  | Mtawanya |
|  |  |  |  | Ngulakula |
|  |  |  |  | Umwe center |
|  |  |  |  | Umwe north |
|  |  |  |  | Umwe south |
|  |  |  | Chumbi | Chumbi A |
|  |  |  |  | Chumbi C |
|  |  |  |  | King‘ongo |
|  |  |  |  | Kiwanga |
|  |  |  |  | Muhoro Magharibi |
|  |  |  |  | Muhoro Mashariki |
|  |  |  |  | Ndundutawa |
|  |  |  |  | Shela |

Supplemental Table 2. Random distribution of missing data.

| Missing Variable | | | Number(n) | Percent (%) |
| --- | --- | --- | --- | --- |
| Member age  (n=9,948) | Fever  (n=176) | Seek treatment  (n=6) |  |  |
| Yes | Yes | Yes | 0 | 0.00 |
| Yes | No | No | 9,906 | 97.79 |
| Yes | Yes | No | 36 | 0.36 |
| Yes | No | Yes | 1 | 0.00 |
| No | Yes | No | 140 | 1.38 |
| No | No | Yes | 5 | 0.05 |
| No | Yes | Yes | 42 | 00.41 |
| Total | | | 10,130 | 100 |

Supplemental Table 3. Results of all baseline covariates between the intervention and control groups, both before and after the application of IPTW weights.

| Variable | | Control | | Intervention | |
| --- | --- | --- | --- | --- | --- |
|  |  | proportion weighted (%) | t value | proportion weighted (%) | t value |
| Member age | Under 25 | 48.3*** | 69.9 | 47.2*** | 63.35 |
|  | 25-50 | 29.7*** | 46.48 | 29.8*** | 44.22 |
|  | 50-75 | 17.0*** | 33.08 | 17.8*** | 30.89 |
|  | 75 or above | 4.9*** | 16.83 | 5.3*** | 15.47 |
| Member sex | male | 55.8*** | 114.61 | 55.7*** | 107.91 |
|  | female | 44.2*** | 90.66 | 44.3*** | 85.95 |
| Education of the head-of-household | None | 30.7*** | 68.08 | 31.9*** | 65.77 |
|  | Received education | 69.3*** | 153.62 | 68.1*** | 140.14 |

* p<0.05, ** p<0.01, *** p<0.001

Supplemental Table 4. Results of all Model average marginal effects of intervention and time with 95%CI.

|  | Useful LLINs | | | Sleeping under net | | | Antimalaria drags | | |
| --- | --- | --- | --- | --- | --- | --- | --- | --- | --- |
|  | Coefficient  (95%CI) | standard error | z | Coefficient  (95%CI) | standard error | z | Coefficient  (95%CI) | standard error | z |
| Model 1 | 0.72  (0.69-0.74)^***^ | 0.01 | 62.68 | 0.88  (0.86-0.89)^***^ | 0.09 | 98.99 | 0.33  (0.19-0.47)^***^ | 0.07 | 4.61 |
| Model 2 | 0.71  (0.69-0.73)^***^ | 0.01 | 60.23 | 0.88  (0.86-0.90)^***^ | 0.09 | 100.99 | 0.33  (0.19-0.46)^***^ | 0.07 | 4.60 |
| Model 3 | 0.70  (0.68-0.73)^***^ | 0.01 | 59.00 | 0.88  (0.86-0.89)^***^ | 0.09 | 99.20 | 0.32  (0.19-0.46)^***^ | 0.07 | 4.62 |
| Model 4 |  |  |  | 0.87  (0.85-0.89)^***^ | 0.1 | 90.88 | 0.37  (0.22-0.51)^***^ | 0.07 | 5.01 |

* p<0.05, ** p<0.01, *** p<0.001

Model 1 unadjusted, Model 2 adjusted Wealth index, Model 3 adjusted Wealth index and household, Model 4 adjusted Wealth index, household and respondent.

Supplemental Table 5. Characteristics of participants, households in the baseline survey.

| **Variable** |  | **Baseline survey (N=9,552, %)** | | | **χ2** | **p-value** |
| --- | --- | --- | --- | --- | --- | --- |
|  |  | **Control** | **Intervention** | **Total** |  |  |
|  |  | **(N=4,871, %)** | **(N=4,681, %)** | **(N=9,552, %)** |  |  |
| **Household** | | | | | | |
| Education of the head-of-household | None | 1,940 (39.83) | 1,853 (39.59) | 3,793 (39.71) | 0.0584 | 0.809 |
|  | Received education | 2,931 (60.17) | 2,828 (60.41) | 5,759 (60.29) |  |  |
| Wealth index | 1^st^ tertile | 1,795 (36.85) | 1,798 (38.41) | 3,658 (38.04) | 23.234 | <0.001 |
|  | 2^ed^ tertile | 1,704 (34.98) | 1,427 (30.48) | 3,131 (32.56) |  |  |
|  | 3^rd^ tertile | 1,372 (28.17) | 1,456 (31.10) | 2,828 (29.41) |  |  |
| Net ownership | Yes | 3,395 (69.70) | 3,929 (83.94) | 7,324 (76.68) | 270.530 | <0.001 |
|  | No | 1,476 (30.30) | 752 (16.06) | 2,228 (23.32) |  |  |
| Useful LLINs | Yes | 2,000 (41.06) | 2,627 (56.12) | 4,627 (48.44) | 216.801 | <0.001 |
|  | No | 1,395 (58.94) | 2,054 (43.88) | 4,925 (51.56) |  |  |
| Sleeping under net (n=4,627) | Yes | 1,032 (51.60) | 1,744 (66.39) | 2,776 (60.00) | 103.456 | <0.001 |
|  | No | 968 (48.40) | 883 (33.61) | 1,851 (40.00) |  |  |
| **Respondent** | | | | | | |
| Gender of the respondent | Male | 2,699 (55.41) | 2,508 (53.58) | 5,207 (54.51) | 3.228 | 0.072 |
|  | Female | 2,172 (44.59) | 2,173 (46.42) | 4,345 (45.49) |  |  |
| Age of the respondent (n=6,398) | Under 25 | 1,850 (53.98) | 1,564 (52.64) | 3,414 (53.36) | 9.239 | 0.026 |
|  | 25-50 | 844 (24.63) | 823 (27.70) | 1,667 (26.06) |  |  |
|  | 50-75 | 577 (16.84) | 470 (15.82) | 1,047 (16.36) |  |  |
|  | 75 or above | 156 (4.55) | 114 (3.84) | 270 (4.22) |  |  |
| Fever (n=9,376) | Yes | 431 (9.01) | 635 (13.83) | 1,066 (11.37) | 54.110 | <0.001 |
|  | No | 4,354 (90.99) | 3,956 (86.17) | 8,310 (88.63) |  |  |
| Seeking treatment (n=1,061) | Yes | 298 (69.30) | 426 (67.51) | 724 (68.24) | 0.378 | 0.539 |
|  | No | 132 (30.70) | 205 (32.49) | 337 (31.76) |  |  |
| Antimalaria drags (n=724) | Yes | 140 (46.98) | 277 (65.02) | 417 (57.60) | 23.374 | <0.001 |
|  | No | 158 (53.02) | 149 (34.98) | 307 (42.40) |  |  |

Supplemental Table 6. Characteristics of participants, households in the endline survey.

| **Variable** |  | **Endline survey (N=10,134, %)** | | | **χ^2^** | **p-value** |
| --- | --- | --- | --- | --- | --- | --- |
|  |  | **Control** | **Intervention** | **Total** |  |  |
|  |  | **(N=5,574, %)** | **(N=4,560, %)** | **(N=10,134, %)** |  |  |
| **Household** | | | | | | |
| Education of the head-of-household | None | 1,280 (22.96) | 1,089 (23.88) | 2,369 (23.38) | 1.180 | 0.277 |
|  | Received education | 4,294 (77.04) | 3,471 (76.12) | 7,765 (76.62) |  |  |
| Wealth index | 1^st^ tertile | 1,572 (28.20) | 1,441 (31.60) | 3,013 (29.73) | 16.364 | <0.001 |
|  | 2^ed^ tertile | 1,967 (35.29) | 1,474 (32.32) | 3,441 (33.96) |  |  |
|  | 3^rd^ tertile | 2,035 (36.51) | 1,645 (36.07) | 3,680 (36.31) |  |  |
| Net ownership | Yes | 5,141 (92.23) | 4,161 (91.25) | 9,302 (91.79) | 3.208 | 0.073 |
|  | No | 433 (7.77) | 399 (8.75) | 832 (8.21) |  |  |
| Useful LLINs | Yes | 3,842 (68.93) | 3,052 (66.93) | 6,894 (68.03) | 4.600 | 0.032 |
|  | No | 1,732 (31.07) | 1,508 (33.07) | 3,240 (31.97) |  |  |
| Sleeping under net (n=6,894) | Yes | 3,214 (83.65) | 2,556 (88.01) | 5,900 (85.58) | 26.125 | <0.001 |
|  | No | 628 (16.35) | 366 (11.99) | 994 (14.42) |  |  |
| **Respondent** | | | | | | |
| Gender of the respondent | Male | 3,229 (57.93) | 2,545 (55.81) | 5,774 (56.98) | 4.591 | 0.032 |
|  | Female | 2,345 (42.07) | 2,015 (44.19) | 4,360 (43.02) |  |  |
| Age of the respondent (n=3,340) | Under 25 | 702 (38.94) | 539 (35.07) | 1,241 (37.16) | 5.958 | 0.114 |
|  | 25-50 | 651 (36.11) | 575 (37.41) | 1,226 (36.71) |  |  |
|  | 50-75 | 336 (18.64) | 311 (20.23) | 647 (19.37) |  |  |
|  | 75 or above | 114 (6.32) | 112 (7.29) | 226 (6.77) |  |  |
| Fever | Yes | 533 (9.56) | 373 (8.18) | 906 (8.94） | 5.888 | 0.015 |
|  | No | 5,041 (90.44) | 4,187 (91.82) | 9,228 (91.06) |  |  |
| Seeking treatment (n=905) | Yes | 315 (59.10) | 229 (61.56) | 544 (60.11) | 0.553 | 0.457 |
|  | No | 218 (40.90) | 143 (38.44) | 361 (39.89) |  |  |
| Antimalaria drags (n=544) | Yes | 58 (18.41) | 22 (9.61) | 80 (14.71) | 8.197 | 0.004 |
|  | No | 257 (81.59) | 207 (90.39) | 464 (85.29) |  |  |

Supplemental Table 7. Regression results of association between household wealth index and Household malaria prevention, malaria treatment during 1,7-mRCTR.

| **Model** | **Variable** |  | **Useful LLINs** | | **Pseudo R²** | **AIC** | **sleeping under net** | | **Pseudo R** | **AIC** | **malaria drugs** | | **Pseudo R** | **AIC** |
| --- | --- | --- | --- | --- | --- | --- | --- | --- | --- | --- | --- | --- | --- | --- |
|  |  |  | **OR (95%CI)** | **P value** |  |  | **OR (95%CI)** | **P value** |  |  | **OR (95%CI)** | **P value** |  |  |
| **Model 1 unadjusted** | Intervention | Control | Ref |  | 0.03 | 25850 | Ref |  | 0.06 | 11465 | Ref |  | **0.15** | **1340** |
|  |  | Intervention | 1.76 (1.59-1.94) | **<0.001** |  |  | 2.10 (1.80-2.44) | **<0.001** |  |  | 2.98 (1.99-4.48) | **<0.001** |  |  |
|  | Survey wave | Baseline | Ref |  |  |  | Ref |  |  |  | Ref |  |  |  |
|  |  | Endline | 2.64 (2.35-2.97) | **<0.001** |  |  | 4.08 (3.40-4.89) | **<0.001** |  |  | 0.28 (0.15-0.53) | **<0.001** |  |  |
|  | Intervention*Survey wave |  | 0.54 (0.46-0.65) | **<0.001** |  |  | 0.60 (0.46-0.78) | **<0.001** |  |  | 0.26 (0.10-0.68) | **0.006** |  |  |
| **Model 2 adjusted Wealth index** | Intervention | Control | Ref |  | 0.05 | 25245 | Ref |  | 0.08 | 11208 | Ref |  | **0.16** | **1338** |
|  |  | Intervention | 1.78 (1.61-1.97) | **<0.001** |  |  | 2.21 (1.89-2.57) | **<0.001** |  |  | 2.90 (1.92-4.38) | **<0.001** |  |  |
|  | Survey wave | Baseline | Ref |  |  |  | Ref |  |  |  | Ref |  |  |  |
|  |  | Endline | 2.52 (2.24-2.84) | **<0.001** |  |  | 4.32 (3.58-5.22) | **<0.001** |  |  | 0.27 (0.14-0.52) | **<0.001** |  |  |
|  | Intervention*Survey wave |  | 0.47 (0.37-0.62) | **<0.001** |  |  | 0.32 (0.22-0.47) | **<0.001** |  |  | 0.20 (0.04-1.07) | **0.060** |  |  |
|  | Wealth index | 3^rd^ tertile (wealthiest) | Ref |  |  |  | Ref |  |  |  | Ref |  |  |  |
|  |  | 2^nd^ tertile | 0.54 (0.48-0.61) | **<0.001** |  |  | 0.49 (0.41-0.58) | **<0.001** |  |  | 1.07 (0.65-1.76) | 0.791 |  |  |
|  |  | 1^st^ tertile (poorest) | 0.38 (0.34-0.43) | **<0.001** |  |  | 0.39 (0.33-0.47) | **<0.001** |  |  | 0.67 (0.40-1.09) | 0.107 |  |  |
|  | Intervention*Survey wave*Wealth index | 1*3^rd^ tertile (wealthiest) | Ref |  |  |  | Ref |  |  |  | Ref |  |  |  |
|  |  | 1*2^ed^ tertile | 1.06 (0.79-1.42) | 0.688 |  |  | 1.67 (1.08-2.58) | **0.020** |  |  | 1.20 (0.19-7.43) | 0.846 |  |  |
|  |  | 1*1^st^ tertile (poorest) | 1.47 (1.09-1.96) | **0.010** |  |  | 3.31 (2.07-5.30) | **<0.001** |  |  | 1.60 (0.22-11.50) | 0.642 |  |  |
| **Model 3 adjusted Wealth index and household** | Intervention | Control | Ref |  | 0.06 | 25077 | Ref |  | 0.09 | 11167 | Ref |  | 0.16 | 1337 |
|  |  | Intervention | 1.79 (1.62-1.99) | **<0.001** |  |  | 2.22 (1.91-2.60) | **<0.001** |  |  | 2.92 (1.93-4.42) | **<0.001** |  |  |
|  | Survey wave | Baseline | Ref |  |  |  | Ref |  |  |  | Ref |  |  |  |
|  |  | Endline | 2.42 (2.15-2.74) | **<0.001** |  |  | 4.19 (3.48-5.06) | **<0.001** |  |  | 0.26 (0.14-0.50) | **<0.001** |  |  |
|  | Intervention*Survey wave |  | 0.48 (0.37-0.62) | **<0.001** |  |  | 0.33 (0.22-0.48) | **<0.001** |  |  | 0.20 (0.04-1.06) | **0.005** |  |  |
|  | Wealth index | 3^rd^ tertile (wealthiest) | Ref |  |  |  | Ref |  |  |  | Ref |  |  |  |
|  |  | 2^nd^ tertile | 0.58 (0.52-0.66) | **<0.001** |  |  | 0.53 (0.44-0.63) | **<0.001** |  |  | 1.13 (0.69-1.87) | 0.621 |  |  |
|  |  | 1^st^ tertile (poorest) | 0.43 (0.38-0.48) | **<0.001** |  |  | 0.44 (0.36-0.52) | **<0.001** |  |  | 0.75 (0.43-1.26) | 0.270 |  |  |
|  | Intervention*Survey wave*Wealth index | 3^rd^ tertile (wealthiest) | Ref |  |  |  | Ref |  |  |  | Ref |  |  |  |
|  |  | 2^nd^ tertile | 1.04 (0.78-1.39) | 0.795 |  |  | 1.58 (1.02-2.44) | **0.041** |  |  | 1.22 (0.20-7.57) | 0.828 |  |  |
|  |  | 1^st^ tertile (poorest) | 1.45 (1.08-1.94) | **0.014** |  |  | 3.24 (2.02-5.21) | **<0.001** |  |  | 1.54 (0.21-11.21) | 0.669 |  |  |
|  | Education of the head of household | None | Ref |  |  |  | Ref |  |  |  | Ref |  |  |  |
|  |  | Received education | 1.52 (1.39-1.66) | **<0.001** |  |  | 1.39 (1.21-1.59) | **<0.001** |  |  | 1.32 (0.89-1.95) | 0.171 |  |  |
| **Model 4 adjusted Wealth index, household and respondent** | Intervention | Control |  |  |  |  | Ref |  | 0.11 | 10863 | Ref |  | 0.18 | 1312 |
|  |  | Intervention |  |  |  |  | 2.24 (1.91-2.62) | **<0.001** |  |  | 3.37 (2.19-5.19) | **<0.001** |  |  |
|  | Survey wave | Baseline |  |  |  |  | Ref |  |  |  | Ref |  |  |  |
|  |  | Endline |  |  |  |  | 3.79 (3.13-4.60) | **<0.001** |  |  | 0.32 (0.16-0.61) | **0.001** |  |  |
|  | Intervention*Survey wave |  |  |  |  |  | 0.33 (0.22-0.49) | **<0.001** |  |  | 0.20 (0.04-1.07) | **0.060** |  |  |
|  | Wealth index | 3^rd^ tertile (wealthiest) |  |  |  |  | Ref |  |  |  | Ref |  |  |  |
|  |  | 2^nd^ tertile |  |  |  |  | 0.52 (0.43-0.62) | **<0.001** |  |  | 1.13 (0.68-1.88) | 0.631 |  |  |
|  |  | 1^st^ tertile (poorest) |  |  |  |  | 0.44 (0.37-0.52) | **<0.001** |  |  | 0.76 (0.45-1.29) | 0.308 |  |  |
|  | Intervention*Survey wave*Wealth index | 1*3^rd^ tertile (wealthiest) |  |  |  |  | Ref |  |  |  | Ref |  |  |  |
|  |  | 1*2^nd^ tertile |  |  |  |  | 1.63 (1.04-2.54) | **0.031** |  |  | 1.17 (0.18-7.54) | 0.866 |  |  |
|  |  | 1*1^st^ tertile(poorest) |  |  |  |  | 3.14 (1.94-5.08) | **<0.001** |  |  | 1.58 (0.22-11.58) | 0.652 |  |  |
|  | Education of the head of household | None |  |  |  |  | Ref |  |  |  | Ref |  |  |  |
|  |  | Received education |  |  |  |  | 1.41 (1.22-1.63) | **<0.001** |  |  | 1.13 (0.76-1.69) | 0.551 |  |  |
|  | Age of the respondent | Under 25 |  |  |  |  | Ref |  |  |  | Ref |  |  |  |
|  |  | 25-50 |  |  |  |  | 2.09 (1.79-2.44) | **<0.001** |  |  | 0.60 (0.38-0.96) | **0.032** |  |  |
|  |  | 50-75 |  |  |  |  | 1.73 (1.42-2.11) | **<0.001** |  |  | 0.41 (0.22-0.73) | **0.002** |  |  |
|  |  | 75 or above |  |  |  |  | 1.27 (0.91-1.78) | 0.160 |  |  | 0.19 (0.05-0.72) | **0.015** |  |  |
|  | Gender of the respondent | Female |  |  |  |  | Ref |  |  |  | Ref |  |  |  |
|  |  | Male |  |  |  |  | 0.62 (0.54-0.70) | **<0.001** |  |  | 0.93 (0.63-1.38) | 0.719 |  |  |
